# Estimation of the effect of atezolizumab plus bevacizumab on pulmonary arterial hypertension using computed tomography in HCC patients

**DOI:** 10.1038/s41598-023-38377-2

**Published:** 2023-07-17

**Authors:** Takayuki Kondo, Kisako Fujiwara, Miyuki Nakagawa, Kentaro Fujimoto, Sae Yumita, Takamasa Ishino, Keita Ogawa, Terunao Iwanaga, Keisuke Koroki, Hiroaki Kanzaki, Masanori Inoue, Kazufumi Kobayashi, Soichiro Kiyono, Masato Nakamura, Naoya Kanogawa, Sadahisa Ogasawara, Shingo Nakamoto, Tetsuhiro Chiba, Jun Kato, Naoya Kato

**Affiliations:** 1 Department of Gastroenterology, Graduate School of Medicine, Chiba University, 1-8-1, Inohana, Chuo-ku, Chiba, 260-8670 Japan; 2 Ultrasound Center, Chiba University Hospital, Chiba, Japan

**Keywords:** Gastroenterology, Hepatology

## Abstract

The effect of the combination of atezolizumab and bevacizumab (Atez/Bev) for hepatocellular carcinoma (HCC) on pulmonary arterial hypertension (PAH) is unknown. Estimation of PAH by using computed tomography (CT) has recently been proposed. Thus, we aimed to estimate the effect of Atez/Bev on PAH using CT. Altogether, 113 patients who received Atez/Bev for HCC were enrolled. Probable PAH was defined as the diameter of the main pulmonary artery (mPA-D) ≥ 33 mm, whereas suspicious PAH was defined as mPA-D ≥ 29 mm or mPA-D/the diameter of the ascending aorta (aAo-D) ≥ 1.0. Before treatment, probable/suspicious PAH were diagnosed in 7 (6.7%)/22 (21.0%) patients, respectively. mPA-D and mPA-D/aAo-D significantly increased after induction of Atez/Bev. The increment of mPA-D was correlated with the occurrence of post-treatment respiratory/heart failure. In analysis of 55 patients who underwent CT at 3 months after the last dose of Atez/Bev, mPA-D and mPA-D/aAo-D significantly decreased. However, in the group with continuous treatment of other molecular-targeted drugs after Atez/Bev, mPA-D and mPA-D/aAo-D showed no significant change. In conclusion, PAH may not be a rare complication in patients with HCC and should be managed carefully because of the possible negative effect of Atez/Bev on PAH.

## Introduction

Pulmonary arterial hypertension (PAH) is a severe and progressive disease that is characterized by obliteration of pulmonary arteries, which leads to increased pulmonary vascular resistance and subsequent right heart failure^1–3^. Portopulmonary hypertension (PoPH) is a subgroup in the clinical classifications of PAH^4^, and defined as PAH that is a consequence of portal hypertension often caused by liver diseases^5,6^.

Hepatocellular carcinoma (HCC), which is the most common type of primary liver cancer, is among the leading causes of cancer-related deaths worldwide^7^. Recently, the combination of atezolizumab, a fully humanized anti-programmed death-ligand 1 antibody, and bevacizumab, a vascular endothelial growth factor (VEGF) targeting antibody, (Atez/Bev) has been approved as the standard first-line systemic therapy for HCC, based on the IMbrave 150 trial^8,9^.

Bev selectively binds to VEGF and inhibits angiogenesis and neovascularization, leading to the control of cancer cell proliferation and prevention of metastases^10^. VEGF and its tyrosine kinase receptor, VEGF receptor-2, play a central role in angiogenesis, protection of endothelial cell, and destabilization of endothelial barrier function of many respiratory disorders including PAH^1,11^. Contradictory reports suggest both protective and deleterious mechanisms on PAH by inhibition of VEGF signaling. VEGF blockade leads to endothelial cell apoptosis in most organ systems^12^, and the inhibition of VEGF signaling causes PAH by aggravating vascular remodeling^13,14^. Contrarily, VEGF reportedly protects the endothelial cell from injury and apoptosis, decreasing the risk of PAH^15^. Additionally, the use of Atez may promote cytotoxic lymphocytes and affect endothelial functions^16^. The prevalence of PoPH is reported to be 2–6% in patients with portal hypertension and 1–2% in patients with cirrhosis^17–20^. However, the prevalence of PAH in patients with HCC receiving chemotherapy is unknown, and the effect of Atez/Bev on PAH is largely unknown. Since HCC frequently occurs in patients with underlying chronic liver diseases, it is likely that some amount of PAH is present in patients with HCC, and its effect cannot be ignored clinically.

Right heart catheterization (RHC) is the gold standard for PAH assessment. However, it is not frequently performed due to its invasiveness. Recently, measurement of the main pulmonary artery and ascending aorta diameters using computed tomography (CT) has been reported to be useful in PoPH screening^21^. In addition, the variation in the main pulmonary artery size over time is associated with progression in pulmonary arterial pressures and prognosis^22^.

Against these backgrounds, in the present study, we aimed to estimate the prevalence of PAH in HCC patients receiving systemic chemotherapy and to investigate the effect of Atez/Bev on PAH based on the change of pulmonary artery diameter.

## Patients and methods

### Patients

We retrospectively enrolled consecutive patients with HCC who were treated with Atez/Bev between October 2020 and December 2021. Clinical and radiological data were obtained from our institutional database. The treatment plan for HCC was finalized after the benefits and side effects of the various treatment modalities and recommendations from experts were explained. This study set out the following exclusion criteria: (1) patients with missing chest CT data after Atez/Bev administration; (2) patients with pulmonary, cardiogenic, and connective tissue diseases that may affect pulmonary hypertension.

This study conformed to the principles stipulated in the Declaration of Helsinki and was approved by the Ethics Committee of Chiba University Graduate School of Medicine. The Ethics Committee of Chiba University Graduate School of Medicine approved that informed consent was waived because of the retrospective design.

### Definitions

HCC was diagnosed using the American Association for the Study of Liver Disease criteria, and advanced HCC was defined as the presence of macrovascular invasion or extrahepatic metastasis. Portal vein tumor thrombosis (PVTT) was defined as a blood flow abnormality in the portal vein trunk with vascularization assessed by contrast imaging. The clinically recognizable PVTT is VP2-4, with a tumor thrombus above the second-order branches of the portal vein^23^.

### PAH assessment and definition using CT

Chest CT was taken at 5-mm slice thickness. The widest diameter of the main pulmonary artery (mPA-D) and ascending aorta (aAo-D) on the same slice level was measured. The mPA-D was selected within 3 cm of the bifurcation of the pulmonary artery. European guidelines suggest that CT findings of mPA-D ≥ 29 mm or mPA-D/aAo-D ≥ 1.0 might suspect pulmonary hypertension^24^, and Japanese guidelines propose that CT finding of mPA-D ≥ 33 mm might be helpful in the diagnosis of PAH^2^. According to the guidelines and a previous study^2,21,24^, in this study suspicious PAH was defined as the presence of mPA-D ≥ 29 mm or mPA-D/aAo-D ≥ 1.0, whereas probable PAH was defined as the presence of mPA-D ≥ 33 mm.

Chest CT before Atez/Bev administration and after 1 or 2 courses of Atez/Bev was performed. Moreover, chest CT data obtained before and at 3 months after the last dose of Atez/Bev was evaluated. Two experienced hepatologists, blinded to clinical data, measured the mPA-D and aAo-D.

### Statistical analysis

Data are expressed as the mean ± standard deviation for normally distributed continuous variables, median and interquartile range for not normally distributed continuous variables, and frequencies and percentages for categorical variables. Continuous variables were analyzed using Student’s *t*-test, Mann–Whitney *U*-test, or paired *t*-test, as appropriate. Categorical variables were analyzed using Fisher’s exact, chi-squared test, or McNemar’s test, as appropriate. The multivariate analysis was assessed by logistic regression. The cumulative survival rate was calculated using the Kaplan–Meier method. The correlation coefficient (*r*) was assessed using Spearman’s rank correlation coefficient or Pearson correlation coefficient, as appropriate. Statistical data were analyzed using SAS version 9.2 (SAS Institute, Cary, NC), and P < 0.05 was considered statistically significant.

### Consent to Participate (Ethics)

This study conformed to the principles of the Declaration of Helsinki and was approved by the Ethics Committee of Chiba University Graduate School of Medicine. The Ethics Committee of Chiba University Graduate School of Medicine approved that informed consent was waived because of the retrospective design.

## Results

### Patient characteristics

Among the 117 consecutive HCC patients who received Atez/Bev therapy during the study period, 105 patients were enrolled in the analysis after exclusion of 12 patients who met the exclusion criteria (4 patients without chest CT after Atez/Bev administration, 4 patients with atrial fibrillation or chronic heart failure, 2 patients with chronic obstructive pulmonary disease, 2 patients with connective tissue disease). Table 1 summarizes the clinical data of the study population. The median observation period was 8.4 months. The cumulative overall survival rate was 66.0% at 1 year.

**Table 1:** Patient characteristics.

Number of patients	105
Age (years)	74 (69–79)
Sex (male/female)	90/15
Hypertension	72 (68.6%)
Diabetes mellitus	32 (30.5%)
BMI	24.0 (21.5–26.9)
Etiology (HCV/HBV/alcohol/others)	27/16/12/50
Advanced stage HCC	65 (61.9%)
Portal vein tumor thrombosis	22 (21.0%)
Extrahepatic metastasis	46 (43.8%)
Number of HCC lesions > 7	46 (43.8%)
Child–Pugh score	5 (5–6)
Alfa fetoprotein (ng/ml)	33.1 (7.6–608.8)

Data are expressed as mean ± standard deviation, median (interquartile range), or number (%).

HBV, hepatitis B virus; HCC, hepatocellular carcinoma; HCV, hepatitis C virus.

### Assessment of PAH

Before the administration of Atez/Bev, the mean mPA-D, aAo-D, and mPA-D/aAo-D were 26.3 mm, 34.9 mm, and 0.76, respectively. The mPA-D ≥ 29 mm, mPA-D ≥ 33 mm, and mPA-D/aAo-D ≥ 1.0 were found in 21 (20.0%), 7 (6.7%), and 4 (3.8%) patients, respectively. Twenty- wo patients (21.0%) had suspicious PAH, and seven patients (6.7%) were diagnosed as having probable PAH.

### Comparison of clinical findings between patients with suspicious PAH and those with non-suspicious PAH

The mPA-D and mPA-D/aAo-D were significantly larger in patients with suspicious PAH than those with non-suspicious (non-suspicious PAH *vs.* suspicious PAH: 24.9 ± 2.9 *vs.* 31.7 ± 1.9 mm, P < 0.001; 0.72 ± 0.10 *vs.* 0.89 ± 0.11, P < 0.001, respectively). However, there was no significant difference in the aAo-D between the non-suspicious and suspicious PAH (34.6 ± 3.7 *vs.* 36.0 ± 5.1 mm, P = 0.258).

Table 2 shows the results of the univariate analysis for the comparison of clinical findings between suspicious and non-suspicious PAH cases. In the univariate analysis, the percentage of females, alcoholic liver diseases, and diabetes mellitus were significantly higher in patients with suspicious PAH than in those with non-suspicious PAH. Multivariate analysis identified that female sex was a risk factor for suspicious PAH (odds ratio = 7.559 [2.098–27.228], P = 0.002).

**Table 2:** Comparison of clinical findings between non-suspicious and suspicious PAH.

	Non-suspicious PAH (N = 83)	Suspicious PAH (N = 22)	*P* value
Age (years)	75 (69–79)	73 (68–79)	0.984
Female	8 (9.6%)	7 (31.8%)	0.008
Hypertension	54 (65.1%)	18 (81.8%)	0.1322
Diabetes mellitus	21 (25.3%)	11 (50.0%)	0.025
BMI	21.4 (23.5–26.0)	25.2 (22.5–28.7)	0.097
Etiology			
Hepatitis C virus	24 (28.9%)	3 (13.6%)	0.145
Hepatitis B virus	14 (16.9%)	2 (9.1%)	0.367
Alcohol	6 (7.2%)	6 (27.3%)	0.009
Advanced stage HCC	51 (61.5%)	14 (63.6%)	0.851
Portal vein tumor thrombosis	17 (20.5%)	5 (22.7%)	0.818
Extrahepatic metastasis	34 (41.0%)	12 (54.6%)	0.254
Number of HCC lesions > 7	35 (42.2%)	11 (50.0%)	0.510
Treatment history for HCC	29 (34.9%)	9 (40.9%)	0.604
Laboratory data			
Aspartate transaminase (U/L)	50 (30–64)	46 (29–59)	0.373
Alanine aminotransferase (U/L)	35 (25–53)	36 (25–54)	0.560
White blood cell (× 10^9^/L)	6.4 ± 2.3	6.4 ± 1.3	0.815
Bilirubin (mg/dL)	0.9 (0.7–1.2)	0.9 (0.7–0.9)	0.271
Prothrombin time (international normalized ratio)	1.01 (0.96–1.05)	1.02 (0.99–1.05)	0.931
Albumin (g/dL)	3.7 (3.4–4.0)	3.5 (3.3–4.2)	0.623
Creatinine (mg/dL)	0.85 ± 0.21	0.89 ± 0.20	0.523
Platelets (10^9^/L)	119 (162–217)	148 (173–225)	0.665
Alfa fetoprotein (ng/mL)	34.7 (8.5–738.8)	18.3 (5.1–445.6)	0.544
Child–Pugh score	5 (5–6)	6 (5–6)	0.056

Data are expressed as mean ± standard deviation, median (interquartile range), or number (%).

HCC, hepatocellular carcinoma; PAH, pulmonary arterial hypertension.

### Changes in PAH parameters by CT before and after Atez/Bev administration

The median interval of chest CT before and after the administration of Atez/Bev was 1.4 months. The mPA-D and mPA-D/aAo-D significantly increased after the administration of Atez/Bev (pre-Atez/Bev *vs.* post-Atez/Bev: 26.3 ± 3.9 *vs.* 27.5 ± 4.2 mm, P < 0.001; 0.76 ± 0.12 *vs.* 0.79 ± 0.13, P < 0.001, respectively). This was mirrored by an increased number of suspicious and probable PAH cases after Atez/Bev (pre-Atez/Bev *vs.* post-Atez/Bev: 22 (21.0%) *vs.* 31 (29.5%), P = 0.013; 7 (6.7%) *vs.* 13 (12.4%), P = 0.034, respectively) Contrarily, there was no significant difference in the aAo-D after Atez/Bev therapy between the two groups (34.9 ± 4.0 *vs.* 35.1 ± 3.9 mm, P = 0.257). High mPA-D level was the only predictive factor for the group that changed from non-suspicious PAH to suspicious PAH (P < 0.001). The increment of mPA-D was significantly correlated with advanced HCC (r = 0.195, P = 0.046) and older age (r = 0.277, P = 0.004, Table 3). During follow-up, 8 patients (7.6%) had respiratory or heart failure. The increment of mPA-D was correlated with the occurrence of respiratory/heart failure (r = 0.195, P = 0.046) after induction of Atez/Bev, which was associated with a poor prognosis (hazard ratio = 7.389 [2.895–18.860], P < 0.001).

**Table 3:** Correlation of clinical findings with the increment of mPA-D after Atez/Bev administration and the decrease of mPA-D at 3 months after the last dose of Atez/Bev.

	Increment of mPA-D after Atez/Bev administration (mm)		Decrease of mPA-D after the last dose of Atez/Bev (mm)	
	Correlation coefficient (*r*)	*P* value	Correlation coefficient (*r*)	*P* value
Age (years)	0.277	0.004	0.057	0.678
Female	0.057	0.563	0.245	0.072
Hypertension	− 0.004	0.967	0.076	0.580
Diabetes mellitus	− 0.011	0.912	0.014	0.918
BMI	0.095	0.335	− 0.175	0.203
Etiology				
Hepatitis C virus	− 0.189	0.053	− 0.170	0.215
Hepatitis B virus	0.066	0.501	− 0.171	0.212
Alcohol	− 0.037	0.708	− 0.024	0.862
Advanced stage HCC	0.195	0.046	0.035	0.798
Portal vein tumor thrombosis	− 0.010	0.916	− 0.036	0.792
Extrahepatic metastasis	0.115	0.243	− 0.099	0.470
Number of HCC lesions > 7	0.010	0.923	− 0.056	0.688
History of treatment for HCC	− 0.049	0.622	− 0.116	0.401
Laboratory data				
Aspartate transaminase (U/L)	− 0.029	0.772	− 0.038	0.783
Alanine aminotransferase (U/L)	− 0.156	0.113	− 0.239	0.079
White blood cell (× 10^9^/L)	0.034	0.727	0.180	0.189
Bilirubin (mg/dL)	0.022	0.820	0.094	0.493
Prothrombin time (international normalized ratio)	0.012	0.903	0.159	0.246
Albumin (g/dL)	− 0.160	0.103	-0.085	0.536
Creatinine (mg/dL)	− 0.033	0.736	-0.211	0.122
Platelets (10^9^/L)	0.052	0.600	0.113	0.924
Alfa fetoprotein (ng/mL)	− 0.036	0.712	− 0.041	0.767
Child–Pugh score	0.039	0.691	0.098	0.474
Findings on CT before Atez/Bev				
mPA-D (mm)	− 0.067	0.499	− 0.043	0.754
aAo-D (mm)	− 0.006	0.955	0.098	0.477
Suspicious PAH	0.032	0.746	0.066	0.633
PD within 2 months after Atez/Bev	0.012	0.901	− 0.161	0.242
Continuous treatment of other molecular-targeted drugs after completion of Atez/Bev	–	–	− 0.329	0.014
Adverse events of Atez/Bev				
Hypertension	− 0.148	0.131	0.105	0.447
Respiratory/heart failure	0.195	0.046	0.283	0.037

aAo-D, diameter of the ascending aorta; Atez/Bev, atezolizumab/bevacizumab; HCC, hepatocellular carcinoma; mPA-D, diameter of the main pulmonary artery; PD, progressive disease.

### Changes in PAH parameter by CT at 3 months after the last dose of Atez/Bev

In 55 patients who underwent CT at 3 months after the last dose of Atez/Bev, the change in mPA-D and mPA-D/aAo-D between the CT performed during Atez/Bev and that performed at 3 months after the last dose of Atez/Bev. The median interval of chest CT before and after the last dose of Atez/Bev was 3.0 months.

After the last dose of treatment, mPA-D and mPA-D/aAo-D significantly decreased (during Atez/Bev *vs.* 3 months after the last dose of Atez/Bev: 27.8 ± 3.6 mm *vs.* 27.0 ± 3.8 mm, P < 0.001; 0.79 ± 0.11 *vs.* 0.77 ± 0.11, P = 0.002, respectively).The decrease of mPA-D at 3 months after the last dose of Atez/Bev was significantly correlated with the presence of respiratory/heart failure (r = 0.283, P = 0.037) and the absence of continuous treatment of other molecular-targeted drugs after Atez/Bev (r = − 0.329, P = 0.014, Table 3). When stratified into groups with continuous treatment of other molecular-targeted drugs after Atez/Bev and those without, mPA-D and mPA-D/aAo-D showed no significant change in the group with continuous treatment (during Atez/Bev *vs.* 3 months after the last dose of Atez/Bev: 27.9 ± 3.2 *vs.* 27.8 ± 2.8 mm, P = 0.570; 0.76 ± 0.11 *vs.* 0.75 ± 0.11, P = 0.487, respectively). Contrarily, mPA-D and mPA-D/aAo-D significantly decreased in the group without continuous treatment of other molecular-targeted drugs (during Atez/Bev *vs.* 3 months after the last dose of Atez/Bev: 27.8 ± 3.8 *vs.* 26.6 ± 4.1 mm, P < 0.001; 0.80 ± 0.10 *vs.* 0.77 ± 0.12, P = 0.002, respectively).

## Discussion

The assessment of PAH in patients with HCC is important because PAH may not be a rare complication, although PAH has not been noted as an adverse event in the IMbrave 150 trial. In our study, 6.7% of patients with HCC were diagnosed as having probable PAH based on the classification using CT, which is consistent with the results of other published reports^17–20^. To the best of our knowledge, this is the first report showing that Atez/Bev may adversely affect PAH; this adverse effect can be ameliorated by drug discontinuation. Furthermore, this study has identified factors that may contribute to the increment of pulmonary artery diameter after induction of Atez/Bev.

Notably, the number of probable PAH cases based on the CT findings increased up to 12.4% after the induction of Atez/Bev. Treatment with Bev has been reported to increase the risk of many cardiovascular events^25,26^. Reversible cardiomyopathy and left ventricular dysfunction are known as the side effects of Bev, occurring in 2–4% of cases, even in patients without history of cardiac disease^27^. It is also reported that VEGF blockade might cause endothelial cell apoptosis and exacerbates vascular remodeling^12–14^. Drug-induced PAH is an underrecognized potential cause of PAH^28^, accounting for 10.5% of patients in the REVEAL registry^29^, with a prognosis similar to that of other causes of PAH^30^. It has been reported that tyrosine kinase inhibitors (TKIs) may be associated with the development or exacerbation of PAH, and dasatinib, a second-generation tyrosine kinase inhibitor, is being suggested as the causative agent of PAH^28^. The discontinuation of dasatinib and specific PAH therapy may lead to the resolution of PAH^28,31–34^. These are consistent with our results that discontinuation of Atez/Bev was associated with the decrease of mPA-D and mPA-D/aAo-D. Contrarily, continuous treatment using molecularly targeted drugs after the last dose of Atez/Bev therapy interfered with the improvement. These results suggest that VEGF inhibition during Atez/Bev therapy might have a negative effect PAH, and early diagnosis of PAH is important to determine whether Atez/Bev needs to be discontinued or a specific treatment for PAH needs to be added.

Regarding screening for PAH, recent advances in digital technology have improved the reliability of echocardiography-based screening for PAH, and such use of digital imaging has become increasingly popular^20,35–38^. Measurement of the tricuspid regurgitation pressure gradient values by echocardiography is used for the screening of PAH, especially when RHC could not be available^35^. Additionally, CT parameters, mPA-D and mPA-D/aAo-D, has been reported as reliable imaging markers for the screening of PAH, with relatively high sensitivity and specificity^39^. Although RHC is an essential test for the definitive diagnosis of PAH and for determining the efficacy of treatment^35,36^, the imaging-based assessment has significant advantages over RHC, as it is less invasive. In comparison with echocardiography, CT is frequently performed during systemic therapy for HCC; it is one of the essential tools for determining the efficacy of systemic therapy. Therefore, proactive use of CT as the first step modality to select candidate for the further examinations, such as echocardiography and RHC, is advocated because early screening for PAH leads to an early definitive diagnosis in patients with HCC.

It is well known that the idiopathic and familial forms of PAH occur more commonly in women than in men^40,41^. Several studies have recently reported that female sex is related to the risk of pulmonary hypertension, which is consistent with the present results^17,21^. Genetic variation in aromatase and changes in estrogen metabolites have been reported to play an important role in the pathogenesis of PAH.

Interestingly, advanced age and advanced HCC were shown to be factors related to the increment of mPA-D and mPA-D/aAo-D after Atez/Bev treatment. Elderly patients reportedly have a higher likelihood of not being able to complete the trials due to toxicity and a high rate of adverse events when taking TKIs plus PD-1 inhibitors^42^. Therefore, caution should be exercised during the administration of Atez/Bev to elderly patients to avoid the development of side effects. Meanwhile, the latter finding may be explained by tumor-associated cytokines, which lead to further activation of macrophages and intimal proliferation, resulting in exacerbation of PAH^43^. This finding is also supported by data showing a relationship between advanced cancer and portal hypertension^44^. Although tumoral portal hypertension is reported to be a relatively rare condition in patients with HCC^45^, this condition would be a worthwhile area for future research, especially as the prognosis of advanced HCC has improved in recent years^9^.

We expected that hypertension, a side effect of VEGF inhibitors, would cause an increment of aAo-D as well as contribute to the increment of mPA-D. However, hypertension did not contribute to either increment of mPA-D or aAo-D. This may be because the patients in the present study were given calcium channel blockers and/or angiotensin II receptor blockers early in the course of their treatment when the side effects of hypertension appeared.

There are some limitations to our paper. First, the data were retrospectively analyzed at a single center. Therefore, the study is limited in its generalizability due to the possibility of population selection bias. Second, although a definitive diagnosis of PAH is based on echocardiography and RHC, this study has no data on RHC and echocardiography. Therefore, the impact of Atez/Bev on PAH should be examined in a prospective setting, taking into account the data on RHC and echocardiography.

In conclusion, PAH may not be a rare complication in patients treated for HCC, and Atez/Bev treatment may have a possible negative impact on PAH. Moreover, in patients with advanced HCC and elderly patients, the increment of pulmonary artery diameter should be monitored carefully.

## Data Availability

All data generated or analyzed during this study are included in this published article.

## Abbreviations

aAo-D: Diameter of the ascending aorta
Atez/Bev: Atezolizumab/bevacizumab
CT: Computed tomography
HCC: Hepatocellular carcinoma
mPA-D: Diameter of the main pulmonary artery
PAH: Pulmonary arterial hypertension
PoPH: Portopulmonary hypertension
PVTT: Portal vein tumor thrombosis
RHC: Right heart catheterization
TKI: Tyrosine kinase inhibitor
VEGF: Vascular endothelial growth factor
